# An adaptive fusion algorithm for coastal sea altimetry based on dual-frequency Beidou-R carrier phase

**DOI:** 10.1371/journal.pone.0302305

**Published:** 2024-05-09

**Authors:** Jin Xing, Dongkai Yang, Zhibo Zhang, Pengyu Yang, Feng Wang

**Affiliations:** School of Electronic and Information Engineering, Beihang University, Beijing, China; National Research Council, ITALY

## Abstract

This article proposes an integer ambiguity determination method based on Beidou system-reflectometry (Beidou-R) observations of the carrier phase at the B1I and B3I frequencies. To enhance the accuracy of sea surface height (SSH) estimation, this study introduces a parallel filtering algorithm and an adaptive iterative fusion algorithm, enabling data fusion based on the variance at B1I and B3I frequencies. To validate and evaluate the proposed method, a coastal experiment was conducted at the Shenxian River. In this experiment, reflected signals from GEO and IGSO satellites were collected. Data analysis reveals that the method is effective, demonstrating that the root mean square error (RMSE) of SSH achieves 2.85 cm and 2.89 cm for PRN 04 and PRN 33, respectively. Furthermore, the impact of the elevation angle on measurement accuracy is analyzed. This study aims to propose a method to enhance coastal sea surface height estimation, offering potential advancements in sea surface altimetry.

## 1 Introduction

Given the critical challenge posed by rising global mean sea levels due to greenhouse warming, advancing our capability to accurately monitor sea surface height (SSH) becomes imperative [1]. While traditional methods, including tidal gauges (TG) and satellite altimeters, have played a pivotal role in SSH data collection, they encounter limitations regarding spatial coverage, cost, and sensitivity to vertical land motion. This study aims to overcome these challenges by harnessing the capabilities of GNSS-Reflectometry (GNSS-R), specifically through dual-frequency Beidou-R carrier phase measurements, to improve SSH estimation accuracy. Vertical land movement impacts tidal gauge (TG) measurements, necessitating correction through high-precision positioning. Typically, satellite altimeters, as active microwave measuring devices, incur high costs and substantial energy consumption. GNSS-R represents a promising technology for SSH monitoring, capable of enhancing mesoscale ocean current flow observations and retrievals by increasing the spatial density of remote sensing data through ample satellite signals. The altimetric method was originally proposed by Martin-Neira in 1993 [2]. A notable strength of this method is its access to a diverse array of signal sources [3]. Subsequently, SSH measurements have been conducted across various platforms, including spaceborne [4], airborne [5], and ground-based [6].

Despite GNSS-R technology being a promising alternative for SSH monitoring, attributed to its passive nature and extensive signal source availability, it faces distinct limitations. These limitations notably encompass carrier phase ambiguity resulting from signal reflection, significantly impacting accuracy. Resolving integer ambiguity in carrier phase measurements is pivotal to enhancing GNSS-R precision and achieving centimeter-level accuracy in SSH estimation. Employing a collaborative processing approach for direct and reflected signals allows GNSS-R technology to estimate Earth’s surface physical parameters. Reflecting the principles of electromagnetic propagation, signals from Earth’s surface are thought to convey crucial physical parameter information. Examining reflected signals’ characteristic features, such as frequency, phase, amplitude, and polarization mode, enables the inference and mapping of Earth’s surface physical parameters. Over nearly three decades, GNSS-R technology has seen consistent refinement and advancement, becoming a prominent research area in remote sensing worldwide. Compared to traditional ocean exploration methods, GNSS-R technology offers several clear advantages, including a simpler configuration without the need for a transmitter, numerous signal sources, and extensive spatial coverage. Owing to these advantages, GNSS-R technology has garnered growing interest from scholars and research organizations globally.

In 2016, Clarizia achieved GNSS-R sea surface altitude inversion using low-orbit TDS-l satellite data, demonstrating UK-TDS-1 data’s feasibility for altitude inversion and synthesizing 6 months of TDS-1 data to map sea surface heights in the South Atlantic and North Pacific Oceans. The root mean square errors for the inverted sea surface height and the DTU10 model height were 8.1m and 7.4m, respectively, exceeding theoretical error expectations [7]. In December 2016, NASA launched the CYGNSS constellation, comprising eight microsatellites in a 510-kilometer circular orbit, to monitor tropical cyclones with high temporal resolution, accumulating extensive observational data to date [8].

The GNSS-R altimetry method comprises interferometric GNSS-R (iGNSS-R) (which involves cross-correlation of the direct and reflected signals) and conventional GNSS-R (cGNSS-R) (which involves cross-correlation of the reflected signal with a locally generated replica of the transmitted one), as exemplified by CYGNSS [9]. The carrier and C/A code phase can be employed to estimate the SSH in cGNSS-R, relying on the navigation signal structure [10]. While carrier-phase-based methods are capable of attaining centimeter-level precision, code-phase-based methods are characterized by a precision that is limited to meters [11]. However, the latter approach necessitates calm sea conditions. Numerous experiments have demonstrated the necessity of coherent signals for ensuring carrier phase continuity, highlighting the requirement of a low wind speed in the experiment environment [12].

The B3I signal, characterized by a transmission bandwidth of 20.46 MHz, results in a sharper autocorrelation function (ACF) and a higher ranging precision [13]. In recent years, research into coherent signals has surged, attributed to the broad applications of carrier phase altimetry, Mashburn et al. utilized CYGNSS L1 DDM data to enhance the analysis of spaceborne sea surface measurement performance, encompassing accurate retracking of delay, ionospheric delay correction, and orbital error correction. They proposed a retracking method founded on the delay model of the reflected signal, examined high accuracy measurement in coherent/incoherent scenarios, and confirmed the presence of strongly coherent scattered signals in the sea area around Indonesia through CYGNSS [14]. Reference [15] introduces a Kalman filter-based closed-loop carrier phase tracking algorithm employing coherent signals from CYGNSS raw IF measurements to demonstrate centimeter-level carrier phase altimetry. A continuous phase tracking algorithm, proposed for retrieving water levels under rough sea conditions near the Onsala Space Observatory coast, highlights its efficacy in environments with high wind speeds [16]. Regarding carrier phase processing, traditional methods struggle to resolve integer ambiguity from reflected signals due to the reflected signal requiring more travel rounds than the direct signal, thus complicating ambiguity resolution. The advocated integer ambiguity resolution method relies on carrier phase observations from the Beidou system reflectometry at B1I and B3I frequencies.

In comparison to existing sea surface height measurement techniques, our proposed integer ambiguity determination method leverages the unique properties of Beidou’s B1I and B3I frequency signals, markedly enhancing the accuracy and reliability of sea surface height estimation in coastal areas through a parallel filtering algorithm and an adaptive iterative fusion algorithm. This technological innovation expands the application of GNSS-R technology in the field of ocean remote sensing and offers a new solution for high-precision sea surface monitoring in complex coastline environments. Furthermore, the parallel filtering algorithm and adaptive iterative fusion algorithm are recommended to enhance the performance of SSH estimation in real time. The proposed method holds significant potential for sea surface altimetry. This research has the potential to benefit various industries reliant on precise and accurate measurements of sea surface height, including coastal management, navigation, and marine transportation.

Section II of this article outlines the altimetry methodology. Subsequently, carrier phase altimetry results are presented to conclude our investigation. This study introduces a continuous carrier phase altimetry method for water level height measurement. Performance measurement of carrier phase altimetry data, utilizing Beidou dual-frequency B1I and B3I signals, is thoroughly investigated and analyzed.

## 2 Phase altimetry model and proposed method

Firstly, the altimetric model for height retrieval is delineated, followed by an examination of coherent phase observation processing. To achieve integer ambiguity resolution and height retrieval in carrier phase altimetry, the integer ambiguity determination method alongside the adaptive fusion algorithm (AFA) is utilized.

To overcome the challenge of additional propagation path length posed by reflected signals, this study introduces an integer ambiguity resolution method specifically designed for dual-frequency Beidou-R carrier phase measurements. By precisely resolving the integer ambiguity, the precision of SSH estimates is significantly enhanced. This method exploits the unique signal properties at B1I and B3I frequencies to limit the range of possible ambiguities, thus refining the search for the true path length difference and, ultimately, the precise elevation angle. This illustrates that the reflected signal incurs an additional propagation path when compared to the direct one. In the shore-based altimetry experiments conducted in this study, it was found that the propagation paths of the direct and reflected signals in the troposphere are nearly identical, given that the relative height of the receiver is much smaller than the satellite altitude. Thus, in the differential operation where the reflected signal is used to subtract the direct signal, this operation is assumed to effectively cancel out the tropospheric delay bias between the direct and reflected signals. Given this assumption, the tropospheric delay difference is not the focal point of analysis in this study, with the aim of simplifying the data processing flow and focusing on the core optimization part of the method. The LHCP antenna can be envisaged as mirrored below the ground, with its propagation path also reflected in this configuration. Consequently, the mathematical relationship between the height of the LHCP antenna above the sea surface and the propagation path delay of the reflected signal is expressed as follows (in meters):

Δρ=2Hsin(θ) (1)

where Δ*ρ* is the path delay of the reflected signal relative to the direct signal, *H* is the height of the LHCP antenna phase center over the sea surface, and *θ* is the elevation angle of the satellite. To obtain the carrier phase, the initial step involves removing the data bits from the I/Q correlation outputs. The four-quadrant arctan2 function [17] is employed to unwrap the phase, thus eliminating half-cycle jumps in the resulting measurements [18].

The electromagnetic wave scattered from a surface comprises two different components: a coherent component and an incoherent component. Based on the roughness of the sea surface, signals reflected from calm waters demonstrate strong coherence, resulting in a scattered forward field dominated by coherent scattering. In contrast, a rough surface scatters signals omnidirectionally, and both intensity and phase vary according to the scattering angle. The correlation sums *I* and *Q* are provided at the receiver’s output. For continuous phase data collection, initial data filtering is necessary to exclude untracked data. After selecting the available data, a one-second coherent integration is performed with the aim of minimizing instrumental noise errors. Although the proportion of coherent reflection events depends on sea conditions, it remains possible to develop a method capable of retrieving the sea surface height when the coherent scattering component is significant. As indicated in [19], for coherent observations, the phase must meet two conditions: 1) the phase cycle traverses all quadrants of the complex plane in either ascending or descending order, and 2) samples within the phase cycle must be uniformly distributed across all four quadrants.

**Fig 1:**
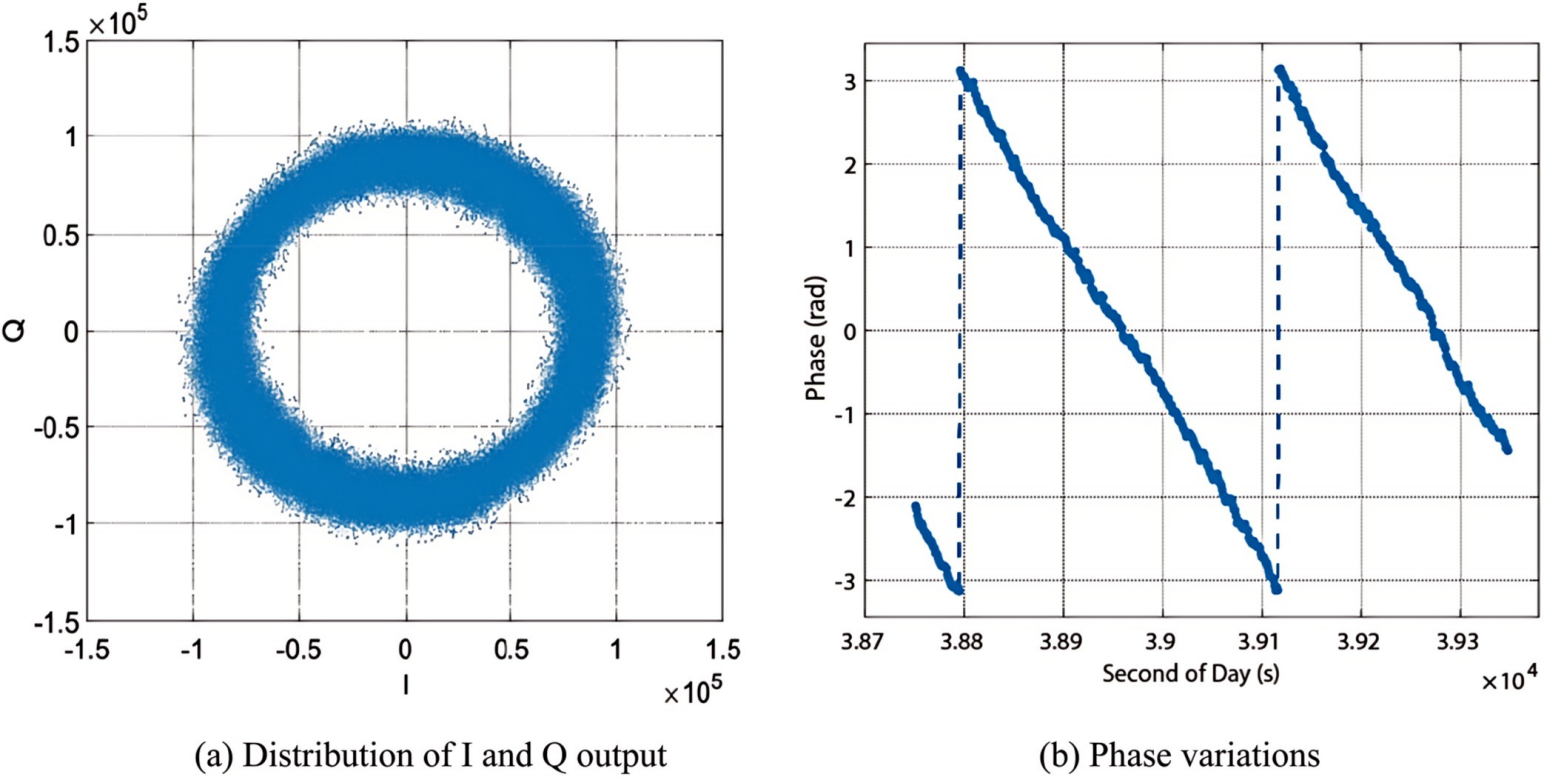
Coherent observations from PRN 34, from 18:40 to 18:50 on November 1st, 2021.

Illustrating coherent observations, Fig 1 showcases the distribution of in-phase and quadrature-phase data, along with the carrier phase obtained between 18:40 to 18:50 local time (UTC+8), on November 1st, 2021. Data were collected from the pseudorandom noise (PRN) 34 satellite of the Beidou system operating at the B1I frequency. Additionally, a definite rotation of the phase occurs consistently within the interval of [-pi, pi].

The relationship between the carrier phase and water level is expressed as follows:

$$h=\frac{(\frac{\varphi }{2\pi }+n)\lambda }{2\sin\theta },$$ (2)

where *h* is the distance between the height of the water surface and the LHCP antenna, *φ* is the carrier phase (the phase difference between the reflected signal and the direct signal), *n* is the ambiguity of the integer cycle, *λ* is the wavelength. B1I signal’ wavelength is 0.1920 m, B3I signal’s wavelength is 0.2363 m, *θ* is the satellite elevation angle.

IGS data are utilized to determine the satellite position at specified times to compute the satellite elevation angle. Given that the receiver transmits only the carrier phase of the reflected signal and not the integer ambiguity *n*, the absolute value of *h* cannot be immediately estimated. Determining the integer ambiguity is essential for retrieving the absolute phase.

Initially, the code phase altimetry method is employed to ascertain the approximate distance range of the water surface. This constrains the ambiguity to a specific range. While the code phase altimetry approach yields meter-level precision, the wider bandwidth of the B3I signal facilitates decimeter-level precision. For this experiment, the 38700th second of the day (SoD) serves as the starting sampling point, with the water level error determined by the code-phase method at this juncture estimated to be within ±1 meter. Based on this, ranges at the B1I and B3I dual frequencies can be separately estimated.

### 2.1 Integer ambiguity determination method

This method employs dual-frequency signals to estimate the integer ambiguity. A single Beidou satellite broadcasts dual-frequency signals, which are simultaneously received by the receiver. Given the identical geometric configuration, the following equations are derived:

$$2[\begin{array}{ccc} \sin\theta & -\lambda_{B1} & 0\\ \sin\theta & 0 & -\lambda_{B3} \end{array}][\begin{array}{l} h\\ n_{B1}\\ n_{B3} \end{array}]=\frac{1}{2\pi }[\begin{array}{l} \lambda_{B1}\varphi_{B1}\\ \lambda_{B3}\varphi_{B3} \end{array}]$$ (3)

where *θ* is the elevation angle of the satellite, *φ*_*B*1_ and *φ*_*B*3_ are the carrier phase observation, respectively. *λ*_*B*1_, *λ*_*B*3_ is the wavelength of the B1I and B3I signals, respectively. *h* is the distance from the water surface to the receiver. *n*_*B*1_ and *n*_*B*3_ are the integer ambiguity at the dual frequency. *h* can be regarded as *h*_*B*1_ and *h*_*B*3_ according to the formula mentioned before.

It is estimated that *h*_*B*1_ and *h*_*B*3_ should be the same at a time. Therefore, by determining the closest height between *h*_*B*1_ and *h*_*B*3_, the linear equation system can be resolved. This paper employs Euclidean and Frechet distances to measure similarity, analyzing continuous water level height curves retrieved under various *n* in the same period, and selects the closest under the two frequencies. Thus, the solution is expressed as:

$$\min\{abs(h_{B3}-h_{B1})\}$$ (4)

With continuous water level monitoring, the carrier phase remains constant once the impact of the satellite’s incident angle on the phase is accounted for. This implies that, after unwrapping the phase, all *n* in the observation time series should be identical. When considering the satellite’s incident angle, a data set exhibits its integer ambiguity. Consequently, a data set’s time series is divided into *i* pieces, each of which generates an *n*. In this paper, all of the *n*_*i*_ in this data set are averaged and are represented as:

$$n_{f}=\langle \bar{\sum \left(n_{i}\right)}\rangle$$ (5)

where 〈⋅〉 stands in for rounding, *n*_*i*_ is the value of *n* at the *i* time, and *n*_*f*_ is the output for the integer ambiguity after being corrected by the integer mean filter.

Therefore, the time series is chosen to ascertain the integer ambiguity using dual-frequency signals, and the distance from the antenna to the water surface is retrieved at the B1I and B3I frequencies, respectively.

### 2.2 Parallel filtering algorithm

In this section, a parallel filtering algorithm is described. Random noise presents a challenge in carrier phase altimetry, prompting the development of a parallel Kalman filter.

Assuming the sea level changes slowly over a short period, a piecewise-linear system is adopted. The rate and acceleration of water level changes are represented by the first and second derivatives, respectively. The state vector is described as:

$$x_{B1/B3}[k]={\left[\begin{array}{llllll} h_{B1}[k] & {\dot{h}}_{B1}[k] & {\ddot{h}}_{B1}[k] & h_{B3}[k] & {\dot{h}}_{B3}[k] & {\ddot{h}}_{B3}[k] \end{array}\right]}^{T}$$ (6)

*k*represents the number of observations in the sequence.

The measurements are modeled as:

$$y_{B1/B3}[k]=Hx_{B1/B3}[k]+v_{B1/B3}[k]$$ (7)

Where **x**_*B*1/*B*3_[*k*] and **v**_*B*1/*B*3_[*k*] the measurement noise, assumed to be white noise. The measurement vector is obtained, and the covariance matrix is described as:

$$R[k]=[\begin{array}{cc} \sigma_{B1}^{2}[k] & 0\\ 0 & \sigma_{B3}^{2}[k] \end{array}]$$ (8)

Here,"−" and "+" superscripts denote prior and posterior Kalman state estimates, respectively. Given the model of water level changes, the state is updated via the following discrete-time model:

$$x^{-}[k+1]=A_{B1/B3}x^{+}[k]$$ (9)

where $A_{B1/B3}=[\begin{array}{ccc} 1 & T & 0.5T^{2}\\ 0 & 1 & T\\ 0 & 0 & 1 \end{array}]$, and *T* is the time interval between measured heights. The state estimation covariance follows the standard propagation step:

$$P^{-}[k+1]=A_{B1/B3P}^{+}[k]A_{B1/B3}^{T}+Q$$ (10)

**Q** represents the system’s prediction covariance. and follow the standard Kalman update steps:

$$K[k+1]=P^{-}[k+1]H^{T}{\left(HP^{-}[k+1]H^{T}+R[k+1]\right)}^{-1}$$ (11)

$$x^{+}[k+1]=x^{-}[k+1]+K[k+1](y[k+1]-Hx^{-}[k+1])$$ (12)

$$P^{+}[k+1]=(I-K[k+1]H)P^{-}[k+1]$$ (13)

### 2.3 Adaptive iterative fusion algorithm

As mentioned above, the parallel filtering algorithm is based on the Kalman filter. However, in a standard Kalman filter, the matrix **R** changes with the measured environment, so it is unreasonable to set **R** to a fixed value. Therefore, this paper designs an adaptive iterative fusion algorithm, which mainly performs the adaptive processing of **R**. Employing iterative variance enhances computational convergence, reduces spatial complexity, suits streaming data, and increases control. Each iteration yields a more precise estimate and reduces errors, saving space by storing only a limited number of variables. Variance estimates can be updated in real-time with the arrival of data streams. Calculation amount and accuracy are flexibly controllable, adapting to various scenarios. To achieve this, a value *r*[*k*] is defined to estimate and implement the adaptive algorithm. For simplicity, corner scales for variables B1 and B3 are omitted.


$$r[k]=\frac{\varphi [k]}{\sin\theta }$$ (14)

Following the altimetry principle, instead of supposing **R**[*k*] as a constant, the variance of the *r*[*k*] relates to the measurement noise. The variance is calculated iteratively as follows:

$$\sigma^{2}[k]=\mathrm{Var}\{r[k]\}=\frac{\tilde{r}[k]}{N}$$ (15)

where *N* is the total sample number, which is used to determine the variance. And $\tilde{r}[k]$ is defined as follows:

$$\tilde{r}[k]=\tilde{r}[k-1]+(r[k]-\bar{r}[k])(r[k]-\bar{r}[k-1])$$ (16)

where the mean of r is

$$\bar{r}[k]=\frac{N-1}{N}\bar{r}[k-1]+\frac{1}{N}r[k].$$ (17)

Once collected independently, dual-frequency data is simultaneously fused with filtering results. The variance at serves as the fusion’s foundation, with weight assigned to it. The result, after weighting, is achieved as follows:

$$h_{\mathrm{fusion}}[k]=\frac{1}{\sigma_{B1}[k]\sigma_{B3}[k]}\left[\sigma_{B1}[k]\;\sigma_{B3}[k]\right]y_{B1/B3}[k]$$ (18)

To compare the subsequent moment with dual-frequency observations, B1I and B3I signals are combined. Filtered observations are then combined using an adaptive filter with weights.

## 3 Experiment and data analysis

### 3.1 Field experiments description

The experiment was conducted utilizing the ground-based phase altimetry method for GNSS-R data collected at the Shenxian River, a tributary of the Yellow River that flows into the Bohai Sea in Dongying city, Shandong, China. The distance between this region and the Bohai Sea, which is directly adjacent, is approximately 2 kilometers. Considerable tidal changes occur in the morning and the evening. This location was identified as suitable for instrument installation, enabling the collection of in-situ data.

**Fig 2:**
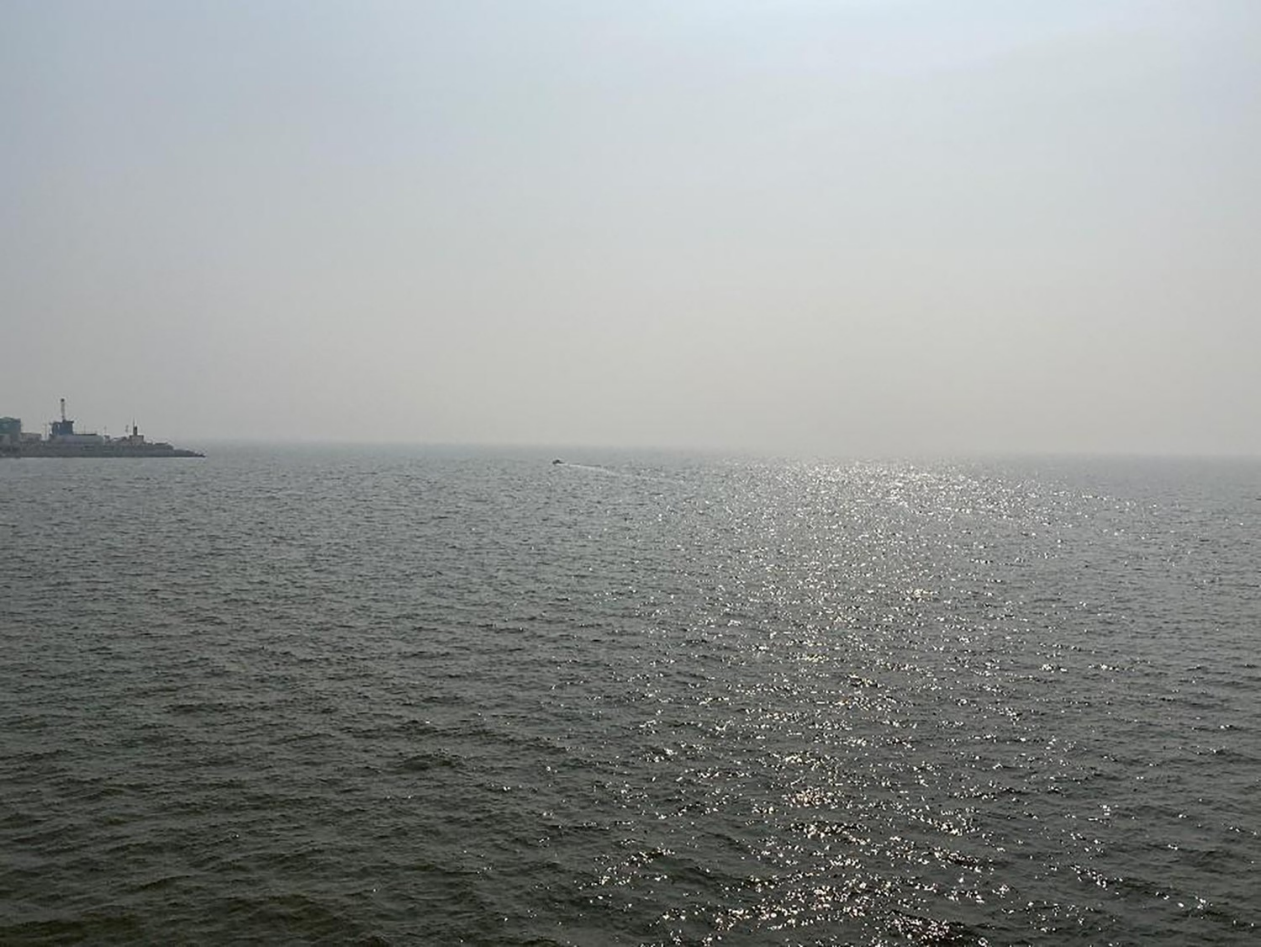
View of the surface water during the experiment.

As illustrated in Fig 2, the sea surface was deemed suitable for the experiment. To avoid multipath signals from the land and trees surrounding the test site, the azimuth range was set from 120° to 270°.

**Fig 3:**
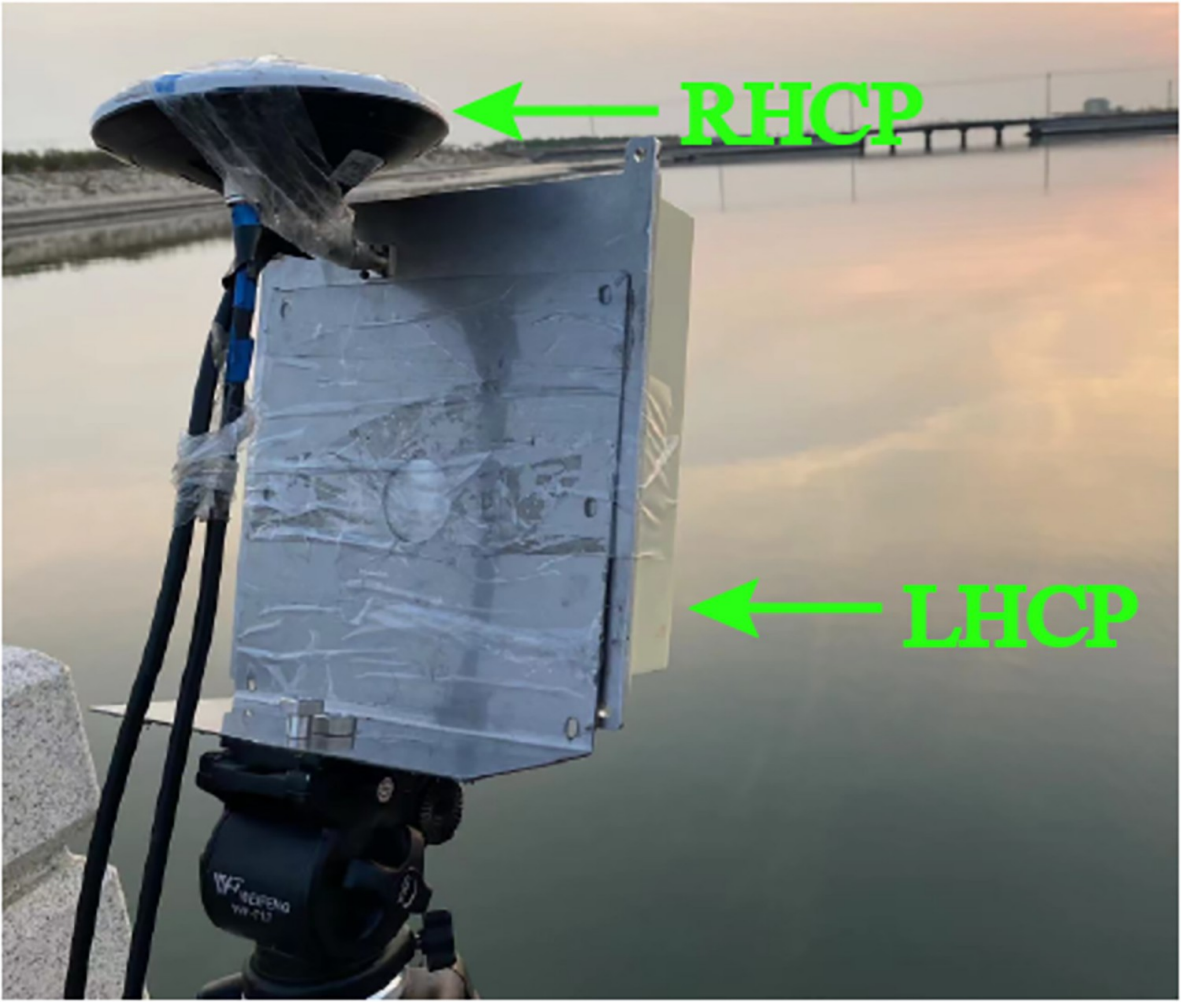
Antenna setup of the experiment.

**Fig 4:**
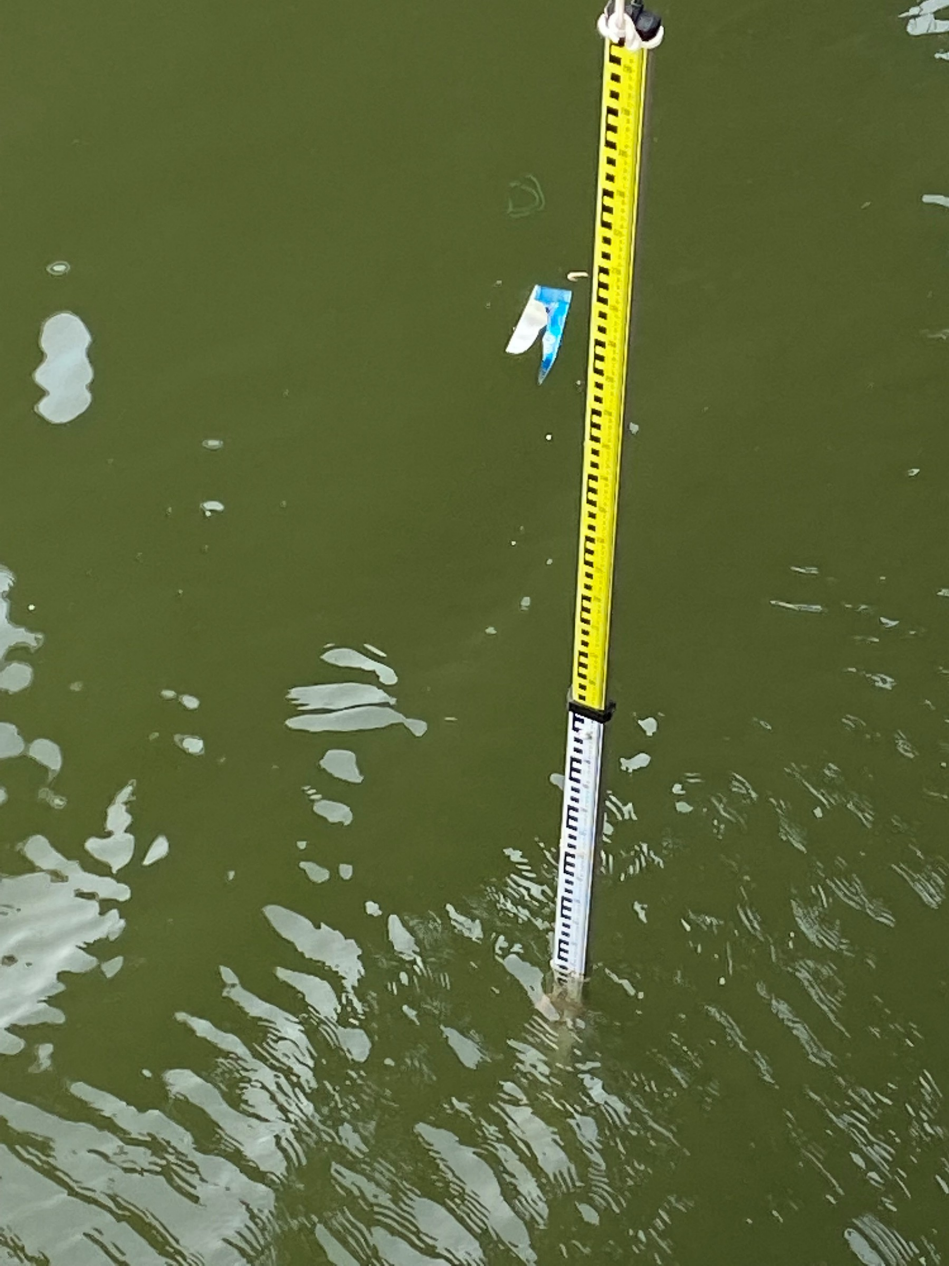
Water ruler setup of the experiment.

The equipment comprises two antennas (LHCP and RHCP), operating at both B1I and B3I frequencies (Fig 3). To acquire in-situ data more precisely and in real-time, the water surface scale was measured manually by positioning a ruler vertically against the water surface, approximately 10 meters away from the receiver (Fig 4).

In the experiment, a four-channel radio-frequency front-end acquisition device was utilized. The device’s intermediate frequency (IF) was set at 0.12 MHz. The sampling frequency for quantizing and sampling the IF analog signal was 32.73 MHz [20–22]. For processing data collected from the Beidou B1I and B3I signals, software was developed. The software includes separate channels for processing direct and reflected signals. To provide a reference for processing reflected signals, direct signals are tracked using a closed-loop phase-locked loop and DLL [23–25]. Subsequently, reflected signals are cross-correlated with a group of local replica signals at various delays to produce the complex delay waveform. By utilizing the fast Fourier transform (FFT) and inverse FFT, parallel cross-correlation in the frequency domain is achieved. In the software, the coherent integration time for B1I and B3I signals is set to 1 ms. Following cross-correlation, the final delay waveform is obtained by incoherently averaging several complex correlation values.

The height from the LHCP antenna to the coastal surface was 2.2 meters, while the distance from the coastal surface to the water surface was approximately 1.4 meters at 18:40, November 1st, 2021 (LT). The distance between the phase centers of the RHCP and LHCP antennas was approximately 15 centimeters. Every 20 minutes, the receiver collects data for a duration of 20 minutes, dividing it into two 10-minute data files. And insitu data is recorded using the ruler every 20 minutes, which is assumed to be a constant in this 20-minute period.

### 3.2 Achieved precision

Using the previously suggested method, we retrieved height data for different satellites. For B1I and B3I frequency observations, we selected the PRN 01 (GEO) and PRN 33 (IGSO) satellites, as discussed in this section. Due to their geometric positioning, GEO satellites provided 12 hours of observation data, while the IGSO satellite yielded only 1200 seconds of data. In both scenarios, we processed a sample every two seconds, thereby acquiring sea surface height data at two-second intervals. We employed spline interpolation and mean smoothing to render the data continuous. We set the average sliding window duration to 60 seconds. By applying the code-phase altimetry method along with preliminary findings, we calculated the water level range to be between 3 and 4.5 meters, and the ranges of *n*_*B*1_ and *n*_*B*3_ towards PRN 01 are calculated to be 19 ~29 and 15~23, according to code-altimetry results. This range facilitates the determination of integer ambiguities. Employing the integer ambiguity determination method proposed in this study, *n*_*B*1_ = 21 and *n*_*B*3_ = 18 can be obtained throughout the observation time period. The water surface height can be retrieved using n that was calculated and the continuous phase carrier of the receiver output. As for PRN 33, *n*_*B*1_ = 16 and *n*_*B*3_ = 13 can be obtained.

**Fig 5:**
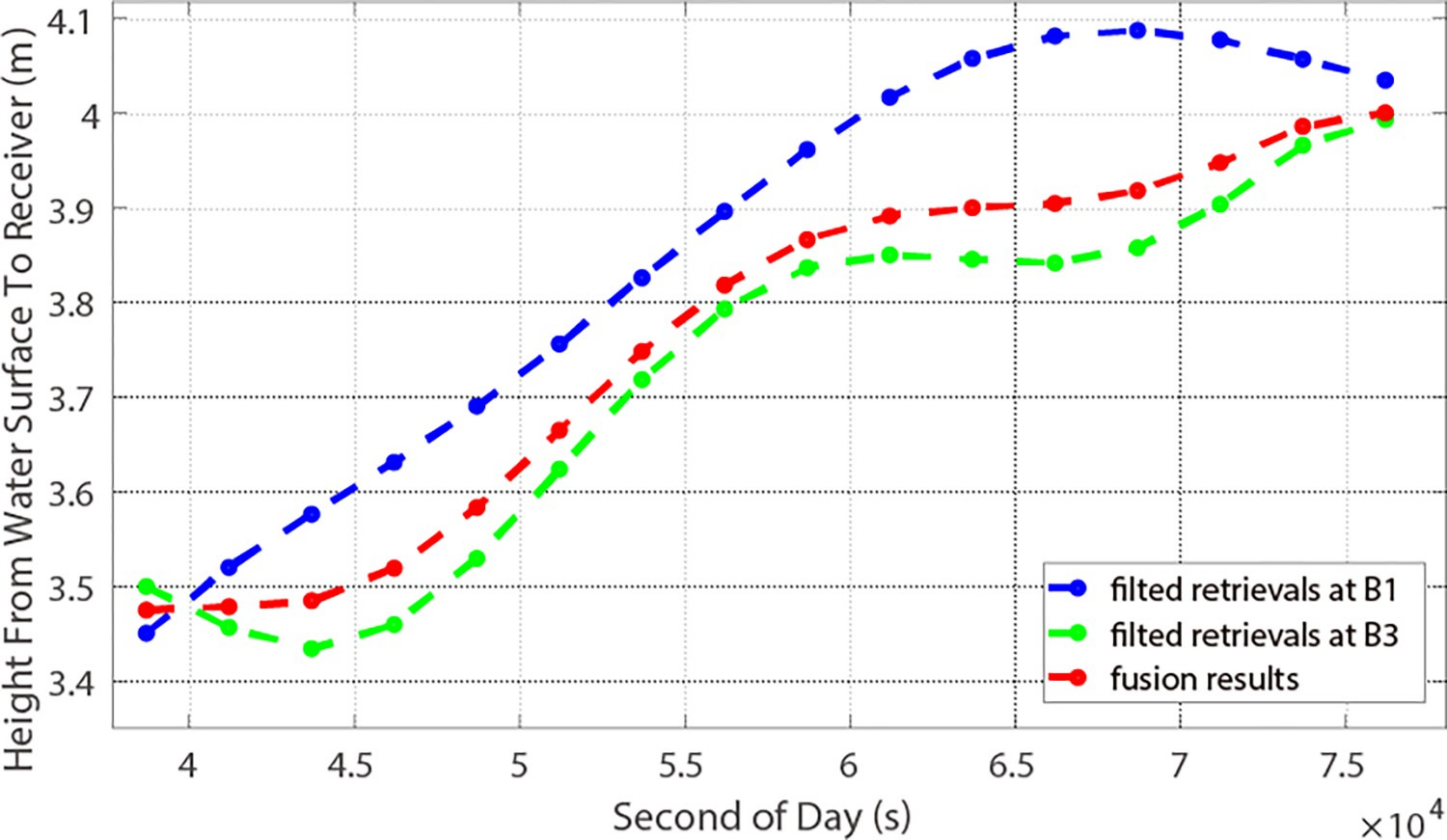
Retrieved height and fusion results using AFA (PRN 01).

**Fig 6:**
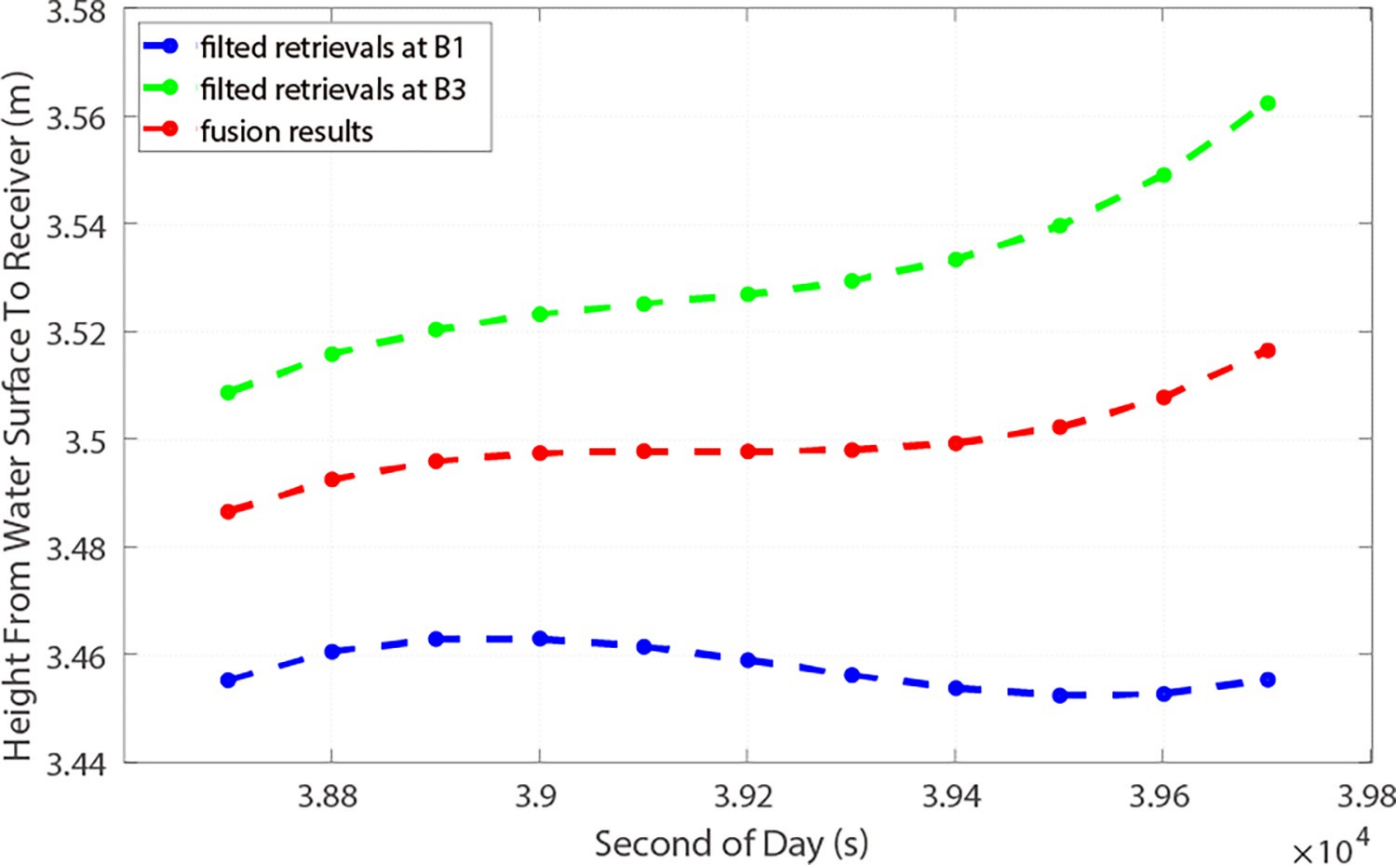
Retrieved height and fusion results using AFA (PRN 33).

PRN 01, a GEO satellite, exhibited height precisions of 10.46 cm and 7.62 cm for the carrier phases at B1I and B3I frequencies, respectively. For a coherent integration time *T*_c_ = 1 ms, and for an incoherent integration time *T*_inc_ = 2 s. With the assistance of the AFA technique, the observation results are illustrated in Fig 5 by the blue and green lines. Notably, the accuracy for B1I and B3I increased to 9.56 cm and 7.02 cm, respectively. The fused data’s accuracy, represented by the red line in Fig 5, was 5.37 cm. Regarding PRN 33, the satellite maintained successful signal reception for 20 minutes, achieving accuracies of 3.68 cm and 6.10 cm at B1I and B3I frequencies, respectively, utilizing the same coherent and incoherent integration times as those for PRN 01. Utilizing AFA, accuracies of 3.38 cm and 5.56 cm were achieved for B1I and B3I, respectively, with the final fusion result displaying an accuracy of 2.89 cm, as depicted in Fig 6.

Table 1 presents the overall accuracy metrics for both satellites. Initially, Table 1 reveals that PRN 33’s accuracy surpasses that of PRN 01, a disparity attributed to environmental factors upon analysis. The GEO satellite undergoes continuous observation for up to 12 hours to monitor the complete tide rising process. Additionally, a 20-minute interval between data files exists, during which external factors, such as wind, could roughen the water’s surface. Consequently, the carrier phase’s accuracy may be compromised. For PRN 33, an IGSO satellite, tracking the phase every second throughout the entire 20-minute observation period contributed to PRN 33’s enhanced results. Across all frequencies, the AFA technique consistently impacts the optimization of retrieved results.

**Table 1:** Accuracy of both cases of PRN 01 and PRN 33.

satelite	B1I	B1I(KF)	B3I	B3I(KF)	B1I&B3I(AFA)
PRN 01	10.46 cm	9.56 cm	7.62 cm	7.02 cm	5.37 cm
PRN 33	3.68 cm	3.38 cm	6.10 cm	5.56 cm	2.89 cm

### 3.3 Accuracy dependence on the elevation angle

**Fig 7:**
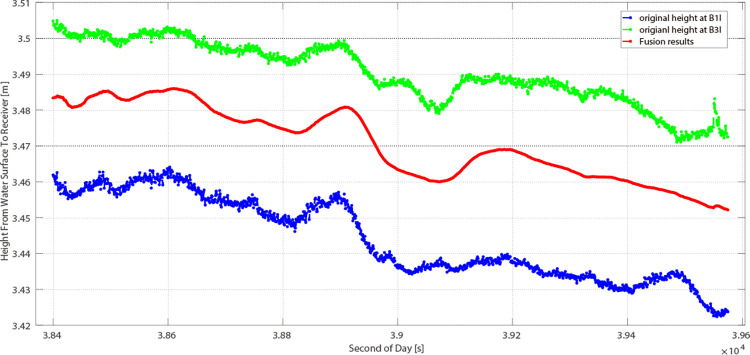
Results from PRN 01 in 20 minutes.

**Fig 8:**
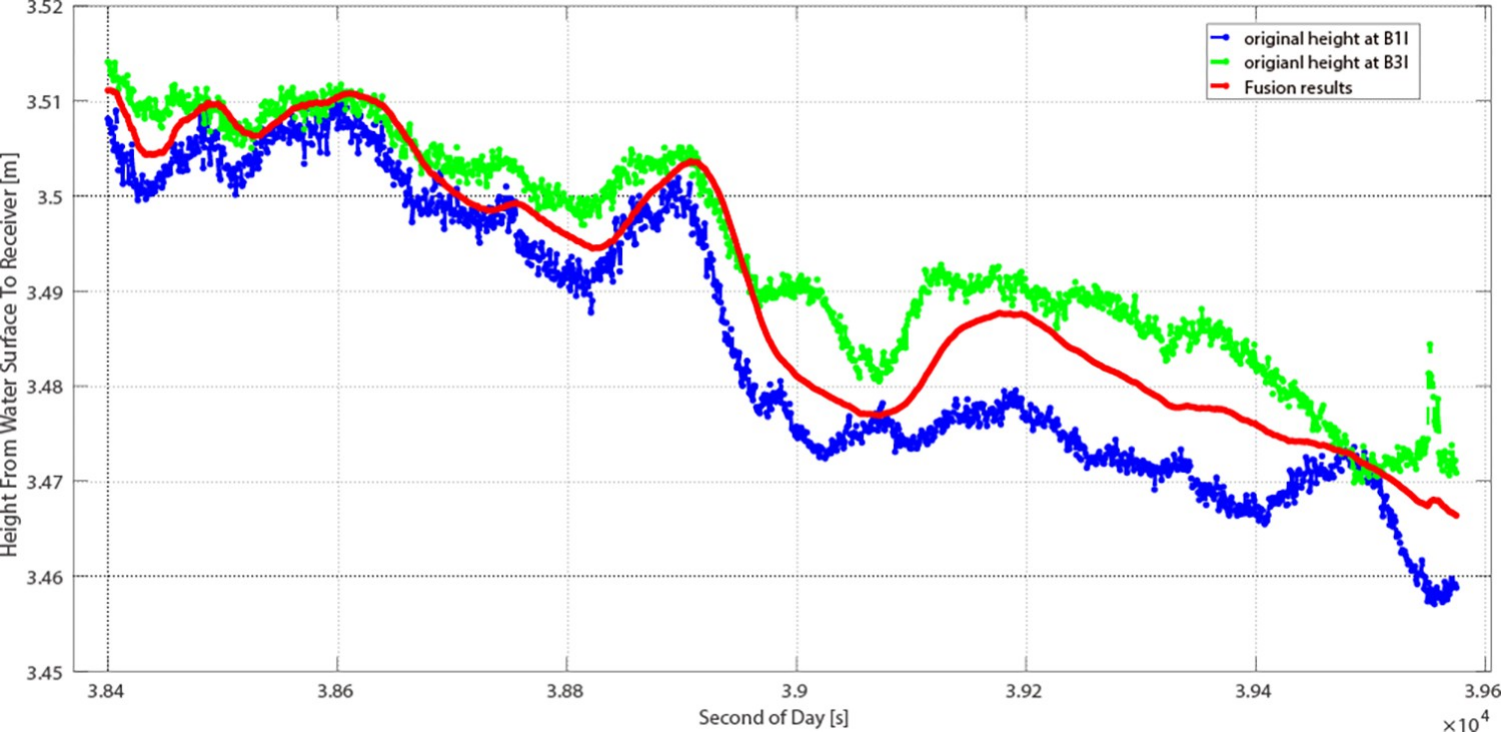
Results from PRN 04 in 20 minutes.

In this study, two GEO satellites were selected. Over the 20-minute observation period, the water level was assumed to be 3.49 meters. The Root Mean Square Error (RMSE) for PRN 01 was 3.60 cm (Fig 7), whereas for PRN 04, it was 2.85 cm (Fig 8). The elevation angles were 38.35° for PRN 01 and 28.66° for PRN 04. This demonstrates that PRN 04’s measurements are more precise across both frequencies. This provides experimental evidence of the relationship between the elevation angle and altimetry precision. It becomes evident that the surface roughness of the reflection is correlated with both the elevation angle and the wavelength of the GNSS signal. A higher satellite elevation angle correlates with increased surface roughness relative to the GNSS signal, enhancing the trackability of the carrier phase [26,27].

In our study, we have demonstrated the precision and accuracy achievable in coastal sea surface height (SSH) measurements using dual-frequency Beidou-R carrier phase observations. Specifically, our experiments focused on PRN 01 (GEO) and PRN 33 (IGSO) satellites, showcasing the method’s effectiveness across different satellite types and frequencies.

For PRN 01, the height precision attained with carrier phase observations at B1I and B3I frequencies were 10.46 cm and 7.62 cm, respectively, before applying the Adaptive Fusion Algorithm (AFA). Post-AFA application, the precision improved significantly, reaching 9.56 cm for B1I and 7.02 cm for B3I frequencies. The fusion of these data further enhanced the accuracy, yielding an impressive 5.37 cm precision. PRN 33 displayed even more remarkable results, with initial accuracies of 3.68 cm for B1I and 6.10 cm for B3I frequencies. The application of AFA fine-tuned these figures to 3.38 cm and 5.56 cm, respectively, and the combined fusion result exhibited a precision of 2.89 cm.

Furthermore, the dependence of accuracy on the satellite’s elevation angle was analyzed, revealing that lower elevation angles yielded more accurate SSH measurements. This was illustrated by the results from two GEO satellites within a 20-minute observation period, where the RMSE for PRN 01 was 3.60 cm, and for PRN 04, it was slightly better at 2.85 cm, underscoring the elevation angle’s impact on altimetry precision. Our findings indicate that the dual-frequency Beidou-R carrier phase observations, complemented by sophisticated data processing techniques like AFA, provide a robust method for high-precision SSH measurement. This advancement holds significant promise for enhancing coastal monitoring and management efforts.

## 4 Conclusion and future work

In this study, we meticulously explored the sea surface height measurement method based on dual-frequency Beidou-R carrier phase observations, assessing its accuracy and practicality. Through the analysis of coastal experimental data, this research not only confirmed the effectiveness of the proposed method but also identified key factors affecting measurement precision. These findings are significant for understanding and enhancing the application of GNSS-R technology in sea surface height measurements. Here are the main conclusions of our study and the outlook for future research directions:

1. As previously noted, the retrieval of ocean surface height from various satellites can be achieved through the data obtained from carrier phase altimetry, characterized by steady and continuous carrier phase observation. Our experiments have surpassed our initial expectations. Significant potential exists for the advancement of sea surface altimetry using carrier phase with dual-frequency Beidou B1I and B3I signals, especially in the application of B3I signals.
2. However, the accuracy of the results is significantly affected by the sea surface’s roughness, with centimeter-level altimetry requiring stringent criteria for sea surface conditions. Future research will focus on carrier phase studies under rough sea surface conditions.
3. The analysis of results from various satellites indicates that the RMSE is within the centimeter range. AFA has enhanced the performance of B1I and B3I signals to varying extents. During continuous observations lasting up to 20 minutes, the RMSE values for PRN 33 and PRN 04 can reach 2.89 cm and 2.85 cm, respectively.
4. At low altitude angles, the incoming wave signals from the scattering surfaces tend to be coherent, suggesting that carrier tracking is more advantageous. Future studies will investigate the impact of environmental factors on continuous carrier phase tracking for shore-based sea surface height retrieval in adverse conditions.

## Supporting information

S1 Data (ZIP)
